# Self-reported antiretroviral therapy adherence and viral load in criminal justice-involved populations

**DOI:** 10.1186/s12879-019-4443-z

**Published:** 2019-10-29

**Authors:** William E. Cunningham, Robin M. Nance, Carol E. Golin, Patrick Flynn, Kevin Knight, Curt G. Beckwith, Irene Kuo, Anne Spaulding, Faye S. Taxman, Fredrick Altice, Joseph A. Delaney, Heidi M. Crane, Sandra A. Springer

**Affiliations:** 1 Department of Medicine, Div GIM & HSR, Geffen School of Medicine, UCLA, 911 Broxton Ave, Los Angeles, CA 90024 USA; 2 Department of Health Policy and Management, Fielding School of Public Health, UCLA, 911 Broxton Ave, Los Angeles, CA 90024 USA; 3 Department of Medicine, University of Washington, Seattle, WA USA; 4 Department of Biostatistics, University of Washington, Box 357232, Seattle, WA 98195-7232 USA; 5 Division of General Medicine and Epidemiology, University of North Carolina at Chapel Hill School of Medicine, Gillings School of Global Public Health, 310 Rosenau Hall, CB #7440, Chapel Hill, NC 27599 USA; 6 Institute of Behavioral Research, Texas Christian University, TCU Box 298740, Fort Worth, TX 76129 USA; 7 Department of Medicine, Alpert Medical School of Brown University and The Miriam Hospital, 1125 North Main St, Providence, RI 02904 USA; 8 Department of Epidemiology and Biostatistics, George Washington University Milken Institute School of Public Health, 950 New Hampshire Ave, NW, 7th Floor, Washington, DC, 20052 USA; 9 Rollins School of Public Health, Emory University, 1518 Clifton Road, Atlanta, GA 30322 USA; 10 Department of Criminology, Law & Society, George Mason University, 4087 University Drive 4100 MSN 6D3, Fairfax, VA 22030 USA; 11 Section of Infectious Diseases, AIDS Program, Yale University School of Medicine, 135 College Street, Suite 323, New Haven, CT 06510-2283 USA; 12 Department of Epidemiology, University of Washington, Seattle, WA USA; 13 Collaborative Health Studies Coordinating Center, Box 354922, Building 29, Suite 210, Seattle, WA 98115 USA; 14 Faculty of Medicine, University of Washington, Seattle, WA USA; 15 Harborview Medical Center, 325 9th Ave, Seattle, WA 98104 USA; 16 Department of Internal Medicine, Section of Infectious Disease, Yale AIDS Program, Yale New Haven Hospital, Yale University School of Medicine, 135 College street, Suite 323, 20 York Street, New Haven, CT 06510 USA

**Keywords:** Antiretroviral therapy, Medication adherence, Viral load, Incarceration, Criminal justice-involved populations (5 key words)

## Abstract

**Background:**

Self-reported antiretroviral therapy (ART) adherence measures that are associated with plasma viral load (VL) are valuable to clinicians and researchers, but are rarely examined among groups vulnerable to dropping out of care. One-seventh of all those living with HIV pass through incarceration annually and criminal-justice (CJ) involved people living with HIV (PLH) are vulnerable to falling out of care. We examined the association of self-reported ART adherence with VL in a criminal-justice sample compared to a routine-care sample.

**Methods:**

Samples: We examined data from a multisite collaboration of studies addressing the continuum of HIV care among CjJ involved persons in the Seek, Test, Treat, and Retain cohort. Data pooled from seven CJ- studies (*n* = 414) were examined and compared with the routine-care sample from the Centers for AIDS Research Network of Integrated Clinical Systems’ seven sites (*n* = 11,698).

Measures: In both samples, data on self-reported percent ART doses taken were collected via the visual analogue scale adherence measure. Viral load data were obtained by blood-draw.

Analysis: We examined the associations of adherence with VL in both cohorts using mixed effects linear regression of log-VL, and mixed effects logistic regression of binary VL (≥ 200 copies/mL) outcomes. Interactions by CD4 count and self-reported health status were also tested.

**Results:**

Among the CJ sample, the coefficient for log-VL was − 0.31 (95% CI = − 0.43, − 0.18; *P* < 0.01) and that in the routine-care sample was − 0.42 (95% CI = − 0.45, − 0.38; *P* < 0.01). For the logistic regression of binary detectable VL on 10% increments of adherence we found the coefficient was − 0.26 (95% CI = − 0.37, − 0.14; *P* < 0.01) and in the routine-care sample it was − 0.38 (95% CI = − 0.41, − 0.35; *P* < 0.01). There was no significant interaction by CD4 count level in the CJ sample, but there was in the routine-care sample. Conversely, there was a significant interaction by self-reported health status level in the criminal-justice sample, but not in the routine-care sample.

**Conclusions:**

The visual analogue scale is valid and useful to measure ART adherence, supporting treatment for CJ- involved PLH vulnerable to falling out of care. Research should examine adherence and VL in additional populations.

## Background

A high level of adherence to antiretroviral treatment (ART) is essential for achieving viral suppression among people living with HIV, and is critical for both maintaining their health and preventing HIV transmission to others. Valid, yet practical measures of adherence to ART are needed for studies of intervention efficacy and effectiveness in low-resource settings, and are useful for the clinical care of hard-to-reach populations who have extensive barriers to achieving viral suppression. Real-world, self-reported ART adherence measures that are reliably associated with viral load (VL) measures provide many advantages over medication event monitoring system (MEMS) or pill count, namely increased feasibility of use in busy clinical care settings where pill counts and MEMS have numerous logistical hurdles for routine use, as well as lower costs and more complete data [1, 2]. Self-report data, however, often underestimate real-world adherence and are susceptible to recall and social desirability bias [3, 4]. These weaknesses may be particularly problematic among those with substance use disorders, mental illness, low income or lower education/literacy levels, and/or unstable housing, which are common among criminal justice-involved persons [5–9].

As 1 in 7 people living with HIV cycle through criminal justice settings each year [10], clinicians may benefit from self-reported ART adherence measures that correlate well with viral suppression among the criminal justice-involved persons they may treat. In this population, frequent measurement of VL is challenging, especially among those recently released from criminal justice settings who often are out of clinical care [5, 7, 8, 11]. Self-reported adherence is an important and practical tool to use in HIV care or interventions that help patients to attain VS. It can be used to identify adherence challenges early, before virologic failure is detected using VL testing. Few, if any, previous studies have examined the association of self-reported adherence with plasma VL among criminal justice-involved persons in multiple U.S. sites.

One of the most widely used measures of self-reported adherence is a single-item, 0–100% rating scale, generally called the visual analogue scale (VAS) [12, 13]. It has the advantages of brevity, ease of administration even among low literacy populations, and ease of interpretation. In usual care settings, evidence supports the validity of VAS for measuring ART adherence, and its practicality compared with longer self-report measures or with more objective measures – such as MEMS Caps or unannounced pill counts (UPC) [14–16]. This single-item assessment is also easier and briefer than other self-report measures [13]. The VAS adherence measure has been shown to be associated with MEMS Caps, UPC, and viral suppression in some studies [4, 17], but has not been examined across studies of criminal justice-involved populations in need of HIV care.

Although several factors besides ART adherence can affect viral suppression including persistence on ART [18], genotypic resistance to ART [19, 20], and pharmacokinetics of ART medications [21–23], self-reported adherence should be closely associated with VL level, and very high adherence (> 95%) should predict VS. [20] Also, the degree of correspondence between the adherence measure and VL might vary, depending on clinical factors, such as level of immunosuppression measured by CD4 count and level of self-reported general health status [24]. Little is known, however, about how well self-reported ART adherence measures perform in terms of its association with VL and levels of viral suppression in criminal justice-involved populations.

This study had two main goals. First, we sought to examine rates of ART adherence and viral suppression among criminal justice-involved people living with HIV across seven sites in the U.S. Second; we aimed to examine the association between self-reported ART adherence and VL. Additionally, we explored whether the relationship between adherence and VL was modified by level of CD4 count or self-reported general health status. We hypothesized that higher levels of self-reported ART adherence would be associated with lower levels of VL or with viral suppression (VL < 200 copies/mL) [25]. In addition, we hypothesized that the association between adherence and VL would be stronger among those reporting worse health or having later stage disease (lower CD4 count) because patients with more advanced disease or who have symptomatic HIV are more likely to both non-adhere to ART and have high plasma HIV RNA levels [26, 27]. To address these goals, we examined associations between ART adherence as measured by the VAS and plasma VL level, using harmonized data from multiple criminal justice-involved studies across the U.S. [28]. To provide a normative comparison group, we examined corresponding measures and associations among people living with HIV in the Centers for AIDS Research Network of Integrated Clinical Systems (CNICS) cohort of people living with HIV in routine, ambulatory clinical care across multiple sites in the US.

## Methods

### Design

The current study uses baseline data from seven sites within the Seek, Test, Treat, and Retain (STTR) cohort [29], a large, previously described, multi-study collaboration addressing the continuum of HIV care among criminal justice samples (Table 1). Harmonized data on people living with HIV pooled from seven individual and pooled criminal justice-focused studies in the criminal justice STTR cohort (*n* = 414) were compared with data on people living with HIV pooled from seven CNICS sites (*n* = 11,698) [30]. CNICS is a continuously enrolling cohort study of people living with HIV in routine clinical care for HIV at multiple sites across the US from 1995 to present. Patient-reported outcomes and laboratory measures including VL values were collected prior to visits, directly via tablets or blood draws, respectively, as part of routine clinical care in CNICS [30]. The data evaluated in this manuscript was collected from 2011 to 2015 for the STTR cohort and from 2007 to 2017 for the CNICS cohort as displayed in Table 2.

**Table 1:** Description of Criminal Justice-involved Studies from STTR Cohort [10]

Studies	Study Design & Location	Targeted Participants
CARE + RCT	RCT of CARE+ Corrections intervention; Washington, DC	Aged 18+; HIV-infected; released from the correctional facility or half-way house ≤6 months ago and living in Washington, DC metropolitan community (not a restricted setting, e.g. half-way house) or currently detained in jail with anticipated release to community (not a restricted setting); reading at 8th grade level and English-speaking.
IMPACT	RCT of imPACT intervention vs. standard of care; NC and TX prisons	Aged 18+; HIV-infected with HIV RNA < 400 copies/mL receiving ART who were incarcerated in NC or TX and 3 months prior to release and not convicted of sexual assault, death or serious injury; English-speaking.
LINK LA	RCT of intervention; Los Angeles County Jails, CA	Men and transgender women, aged 18+; HIV-infected; eligible for ART, or on ART; jailed for 5+ days, being released to community; residing in Los Angeles County, CA upon release; English or bilingual Spanish speaking.
NEW HOPE	Double Blind Placebo-controlled RCT of extended-release naltrexone; New Haven, Hartford, Waterbury, CT or Springfield, MA	Aged 18+, HIV-infected, meeting DSM-IV criteria for opioid dependence, within CT & Springfield, MA corrections system and not pending trial for a felony, within 30 days of being released to greater New Haven, Hartford, Waterbury or Springfield areas or 30 days after release; English- or Spanish-speaking, no liver failure or grade IV hepatitis, no active opioid withdrawal, no receipt of methadone or buprenorphine/naloxone for treatment of opioid dependency, no participation in pharmacotherapy trial in the previous 30 days
STRIDE1	RCT of buprenorphine vs. placebo; Washington, DC	Aged 18+; HIV-infected; meeting DSM-IV criteria for opioid dependence; resident of Washington, DC with eligibility for medical entitlements; English- or Spanish-speaking; no current opiate medications for chronic pain conditions or need to be placed on such medications; no current methadone doses over 30 mg/day, no AST and ALT >5x the ULN; no pregnancy or breast-feeding; no liver dysfunction; no suicidal ideation; no participation in pharmacotherapy trial in the previous 30 days.
STRIDE2	Longitudinal cohort study comparing treatment using opioid substitution therapy to no treatment; Washington, DC	Aged 18+; HIV-infected; meeting DSM-IV criteria for opioid dependence; resident of Washington DC with eligibility for medical entitlements; English-speaking.
SUCCESS	Non-randomized pilot study of Strengths-Based case management; Atlanta, GA Jails	Aged 18+; HIV-infected; detained or sentenced in jail or detention center and likely to leave within 6 weeks; no recent participation in randomized trial to improve retention in HIV care; English-speaking.

**Table 2:** Sociodemographic and Clinical Characteristics of People Living with HIV in Criminal Justice Settings (STTR) and in Routine Clinical Care (CNICS) Study Samples^a^

Sample Characteristics	STTR (%) (Range)^b^	CNICS (%)
N	414	11,698
Mean age [SD]	44 [10]	46 [11]
Male	71 (45–100)	84
Race/Ethnicity		
White	13 (0–22)***	48
Black	73 (39–100)***	34
Hispanic	9 (0–60)	14
Completed High School	62 (50–72)	NA
Homeless	36 (0–66)	NA
Drug Use^c^	74 (48–100)***	37
Binge Alcohol Use	35 (13–56)	32
HIV VL ≥200^d^	26 (15–56)***	11
Log (VL + 1) (mean [SD])	4.3 [2.7] ***	3.6 [2.0]
VAS Adherence ≥95%^e^	59 (39–88)***	71
VAS Adherence (mean % [SD])	88 [20] ***	92 [16]
NNRTI-based regimen	31 (29–41)	29
Protease inhibitor (PI)-based regimen	53 (24–60)***	25
Integrase inhibitor-based (INSTI) regimen	5 (3–15)***	22
Combination/Other regimen	11 (9–20)***	23
Included study timeframe	2011–2015^f^	2007–2017

Significant differences in mean values (e.g., age) and proportions between STTR and CNICS characteristics indicated by: * *P* < 0.05, **P < 0.01, ***P < 0.001

^a^Except IMPACT, where the week 2 VL and VAS adherence values were used

^b^Range of values across 7 included criminal justice-involved studies

^c^Includes Cocaine/crack, opiates, methamphetamines, and marijuana

^d^ VL = viral load

^e^VAS = visual analogue scale of adherence - % of ART medications taken in prior 30 days

^f^Date range of baseline data collection

SD = standard deviation

STTR – Seek Test, Treat, Retain NIDA-funded criminal justice-involved harmonized sample

CNICS - Centers for AIDS Research Network of Integrated Clinical Systems

### Study samples

At each study site, baseline interviews and laboratory measures were collected and processed by individual studies and the STTR Coordinating Center harmonized the data. We analyzed and compared cross-sectional, baseline data from criminal justice-involved people living with HIV in the STTR studies, and with people living with HIV in routine care in CNICS. Criminal justice-involved participants included those in custody or released but under community supervision. Of 1189 criminal justice-involved (STTR) participants, 414 individuals had complete data on adherence and VL within 30 days of the adherence reference period. Of 15,740 possible people living with HIV in routine clinical care, 11,698 participants had complete data on adherence, or a VL within 30 days of the adherence reference period.

### Measures

In both groups, self-reported adherence data were measured using the VAS, on a scale of 0–100% of ART doses taken in the preceding 30 days. Self-reported general health status was measured using the first item of the SF-12 instrument (“In general, would you say your health is: …” ) on a 5-point Likert-type scale with response options that ranged from “poor” to “excellent.” Study sites also collected self-reported data on age and gender for use in adjusted analyses. We also collected data on ART regimens – Non-nucleoside Reverse Transcriptase (NNRTI), Protease Inhibitors (PI), integrase strand transfer inhibitor (INSTI) [31], and combination or other ART regimens. Plasma VL and CD4 cell count data were obtained by each STTR study, thus, there was not a uniform assay used to measure them. We analyzed HIV VL as a log transformed (log-VL) continuous measure, or dichotomized as detectable (≥200 copies/mL) vs. undetectable (VL < 200 copies/mL) in accordance with DHHS guidelines [32].

### Analysis

#### Association of VL and ART adherence

We first examined descriptive characteristics of ART adherence and VL, and then assessed the associations of adherence with VL in each individual criminal justice-involved study, and the combined criminal justice sample, which were then compared to the overall, CNICS, routine HIV care dataset. Next, we constructed linear and logistic mixed effects regression models with random intercepts and slopes, adjusted for age using the criminal justice sample data. To determine the robustness of the associations of adherence with VL, we used three distinct approaches often used in adherence research [2, 33, 34]. These models examined in criminal justice-involved persons living with HIV the study-specific and overall associations of: (A) continuous adherence (10% increments) with continuous log-VL; (B) continuous adherence (10% increments) in a logistic regression with binary detectable VL; and (C) optimal adherence levels using the VAS (≥95%) predictor in a logistic regression with binary detectable VL. Because the separate study sites had relatively small sample sizes, we used mixed effects models clustered by site to appropriately pool study samples, while still allowing for the possibility that the adherence coefficient had a different mean value in each study sub-sample. Linear and logistic regressions adjusted for age, which were also conducted separately in the routine care sample for comparison. In both the criminal justice and routine care samples, we tested for possible non-linearity of the association between adherence and VL over the range of adherence scores, using generalized additive models (GAMs) adjusted for age, sex, and study sample. To test whether ART adherence differs by type of regimen used, we examined linear regression of Log VL on adherence, adjusted for age, sex, and study indicator, with main independent variable the ART regimen type in the criminal justice and routine care samples. Interaction *P*-values were computed for the test of whether the regression coefficient for the given ART type is different than that for NNRTI. Again, the routine care sample was analyzed to provide a normative comparison for the size and direction of effects estimated in the criminal justice sample for all analyses.

#### Effect modification

CD4 cell count and self-reported general health status were assessed as possible effect modifiers of the adherence-VL association in both the criminal justice and routine care samples. CD4 count was dichotomized as ≤ 500 vs. > 500+ cells/mm^3^, and self-reported general health status was categorized as high (excellent, very good, good) vs. low (fair, poor). To test whether the associations between adherence and VL differed significantly by CD4 or health status strata, we conducted linear mixed effect regressions of log-VL outcomes on 10% increments of adherence, age, sex, study site, the effect modifier variable of interest (either CD4 or self-reported general health status, depending on the model) and the interaction of adherence*effect modifier variable. A sensitivity analysis using different cut points to define the health status strata (excellent/very good vs. good/fair/poor) was also performed, and because the results did not differ greatly, we present results using the original cut-point.

## Results

### Descriptive characteristics

Comparing 414 criminal justice-involved people living with HIV from STTR with 11,698 persons in routine HIV care from CNICS (Table 2), the criminal justice-involved persons were significantly (all comparisons significant with *P* < 0.001) more likely to have a detectable VL (26% vs. 12%), greater mean log-VL, smaller proportion with ART adherence scores ≥ 95% (59% vs. 70%), and smaller proportions of ART regimens containing either PI (28% vs. 41%) or NNRTI (23% vs. 42%), or INSTI regimens (5% vs. 22%; Table 2). Furthermore, the criminal justice sample was comprised of a greater proportion of Blacks (73% vs. 30%) and a greater proportion of participants with substance use disorders (73% vs. 50%) than the persons in routine HIV care.

### Associations of VAS adherence with viral load

**Fig. 1:**
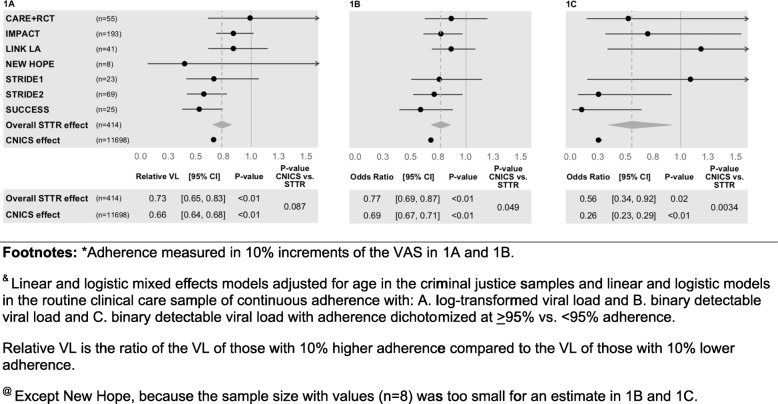
Associations of Adherence* with Viral Load Using Different Parameterizations,^&^ among Criminal Justice-Involved (STTR)^@^ and Routine Clinical Care (CNICS) Study Samples. Footnotes: *Adherence measured in 10% increments of the VAS in **a** and **b**. ^&^ Linear and logistic mixed effects models adjusted for age in the criminal justice samples and linear and logistic models in the routine clinical care sample of continuous adherence with: **a** log-transformed viral load and **b**. binary detectable viral load and **c**. binary detectable viral load with adherence dichotomized at ≥95% vs. <95% adherence. Relative VL is the ratio of the VL of those with 10% higher adherence compared to the VL of those with 10% lower adherence. ^@^ Except New Hope, because the sample size with values (*n*=8) was too small for an estimate in **b** and **c**

We examined the association between VL (both continuous and binary) and adherence (in 10% increments) using mixed effects regression analyses and found, among the criminal justice sample the relative VL was 0.73 (95% CI = 0.65, 0.83; *p* < 0.01; Fig. 1a) indicating that each 10% increment in adherence was associated with a reduction in VL of 27% (1-relative VL%). Similarly in the routine care sample, the relative VL was 0.66 (95% CI = 0.64, 0.68; *p* < 0.01), so each 10% increment in adherence was associated with a reduction in VL of 34%.

**Fig. 2:**
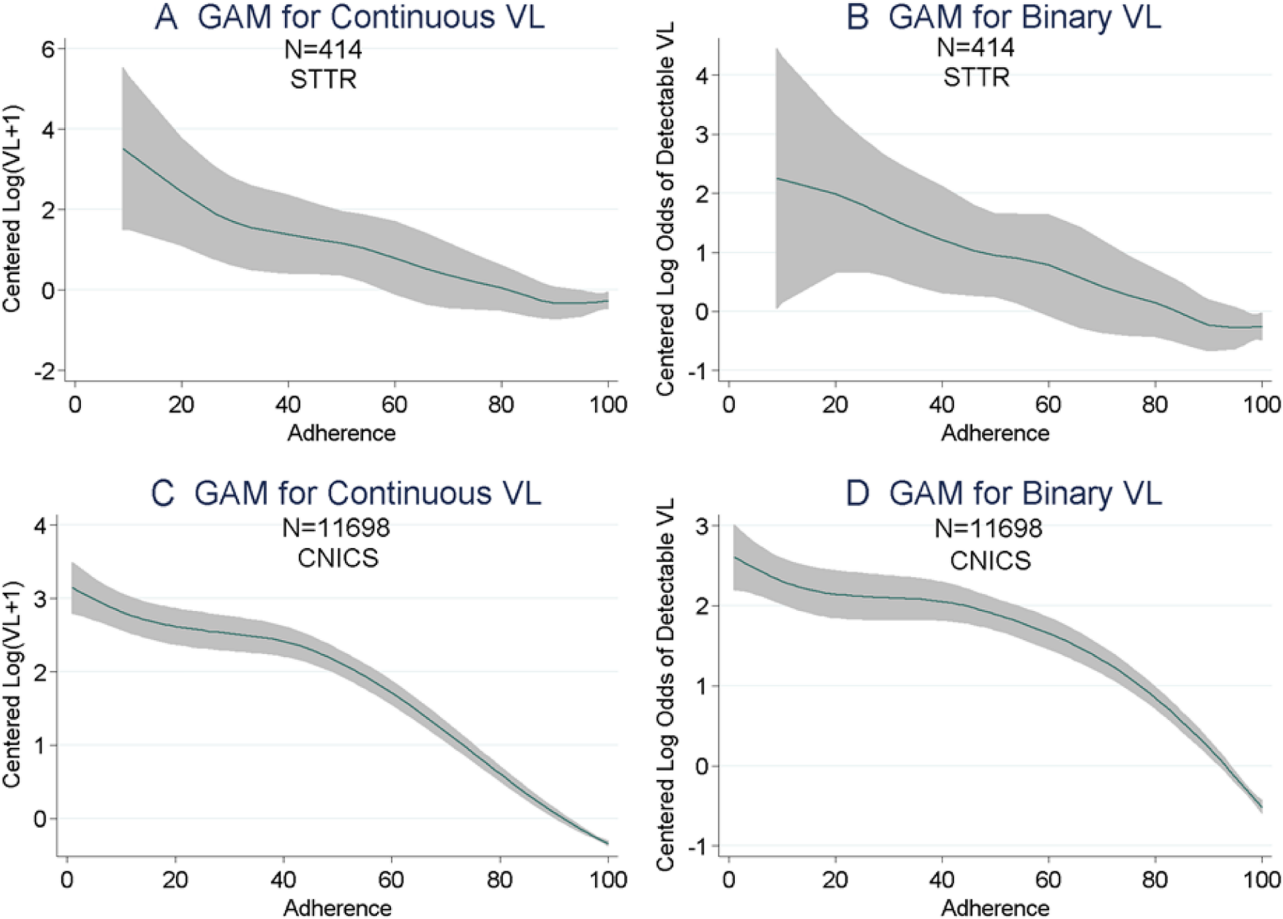
Linearity of Associations between Adherence and Viral Load over the range of Adherence Scores in Criminal Justice-Involved (STTR) Compared with Routine Clinical Care Samples (CNICS)^@^. Footnotes: ^@^ Generalized Additive Model (GAM), adjusted for age and sex, of the Association of Adherence with: **a** Centered Log-VL in the criminal justice sample; **b** Centered Log Odds of Binary VL in the criminal justice sample; **c** Centered log-VL in the routine care sample; and **d** Centered Log Odds of Binary VL in the routine care sample. P-values for A=0.4, B=0.8, C<0.001, D<0.001

The interaction analysis indicated that the coefficients in the two samples were not significantly different from one another (*P* = 0.087). Similarly in the criminal justice sample, for the logistic regression of binary detectable VL on 10% increments of adherence we found an odds ratio (OR) of 0.77 (95% CI = 0.69, 0.87; *P* < 0.01 Fig. 1b), and in the routine care sample it was 0.69 (95% CI = 0.67, 0.71; *P* < 0.01). In this case, however, there was a significant interaction, indicating that the coefficients in the two samples were significantly different from one another (*P* = 0.049). Moreover, we examined the associations of optimal adherence (≥95%) with binary detectable VL, and in the criminal justice sample we found that the OR was 0.56 (95% CI = 0.34, 0.92; *P* = 0.02; Fig. 1c), while in the routine care sample the corresponding OR was 0.26 (95% CI = 0.23, 0.29; *P* = 0.01; *P*-value for the interaction = 0.0034; Fig. 1c). In the criminal justice sample, we further examined the associations of adherence with both continuous log-VL and binary detectable VL using generalized additive models ([GAM]; Fig. 2a & b) and found that the relationship was fairly linear over the range of adherence scores. The GAM analysis also appeared to be approximately linear and with similar slope over the range of adherence scores among people living with HIV in routine clinical care (Fig. 2c & d).

### Potential effect modification of associations of VAS adherence with VL

In the analysis of the association between adherence and VL in the criminal justice sample stratified by CD4 count, we found that the linear coefficients for the regression of Log-VL on 10% VAS adherence, stratified by CD4 count, was − 0.51 (95% CI -0.73, − 0.29; *P* = < 0.001) for those with CD4 < 500; it was − 0.25 (95% CI -0.51, 0.02; *P* = 0.07) for those with CD4 ≥ 500 (Table 3). However, these coefficients were not significantly different from one another in the interaction analysis for the criminal justice sample (*P* = 0.14; Table 3). The corresponding CNICS sample coefficient point estimates, stratified by CD4, were similar to those in the criminal justice sample, but with the much larger sample size in routine care the interaction by CD4 level was significant (*P* < 0.001; Table 3). In the stratified analysis by self-reported general health status in the criminal justice sample, we found that the coefficient for log-VL regressed on adherence was much larger for low health status (− 0.44; 95% CI -0.70, − 0.18; *P* = 0.001) than it was for high health status (0.01; 95% CI -0.25, 0.27; *P* = 0.90) and that the interaction was significant (*P* = 0.01; Table 4). In the routine care sample, the corresponding linear coefficients for the regression of Log-VL on 10% increments of adherence, stratified by self-reported general health status showed no significant interaction by health status (Table 4).

**Table 3:** Associations of Adherence^d^ with Log- viral load, Stratified by CD4 Count Level among Criminal Justice-Involved (STTR) and Routine Clinical Care (CNICS) Study Samples^a^

	Coeff	95% CI	*P*-value	Interaction *P*-value^1^
STTR *N* = 208^b^				
STTR Overall	− 0.42	− 0.62,-0.22	< 0.001	NA
CD4 < 500 (*n* = 129)	− 0.51	− 0.73, − 0.29	< 0.001	0.14
CD4 ≥ 500 (*n* = 79)	− 0.25	− 0.51, 0.02	0.07	Ref
CNICS *N* = 9487^c^				
CNICS Overall	− 0.43	− 0.47, − 0.39	< 0.001	NA
CD4 < 500 (*n* = 4229)	− 0.57	− 0.62, − 0.51	< 0.001	< 0.001
CD4 ≥ 500 (*n* = 5258)	− 0.18	− 0.21, − 0.14	< 0.001	Ref

^**1**^Interaction *P*-value for the test of whether the regression coefficient for CD4 < 500 is different than that for CD4 ≥ 500

^a^Linear regression of Log VL on adherence, adjusted for age, sex, and study indicator, stratified by CD4 count level in the criminal justice and routine care samples

^b^*n* = 206 had missing CD4 values

^c^*n* = 2211 had missing CD4 values

^d^Adherence measured in 10% increments of the VAS

**Table 4:** Associations of Adherence with Log- Viral Load, Stratified by Self-Reported Health Status (HS) in Criminal Justice-Involved (STTR) and Routine Clinical Care (CNICS) Samples^a^

	Coeff	95% CI	*P*-value	Interaction *P*-value^1^
STTR *N* = 196^b^				
STTR Overall	−0.28	− 0.50,-0.07	0.008	NA
Low HS (*n* = 78)	−0.44	−0.70, − 0.18	0.001	0.01
High HS (*n* = 118)	0.01	−0.25, 0.27	0.935	Ref
CNICS *N* = 291^c^				
CNICS Overall	−0.31	−0.51, − 0.11	0.002	NA
Low HS (*n* = 68)	−0.37	−0.62, − 0.11	0.005	0.59
High HS (*n* = 223)	−0.26	−0.56, 0.04	0.084	Ref

^1^Interaction *P*-value tests whether the regression coefficient for CD4 < 500 is different than CD4 ≥ 500

^a^Linear regression of Log-VL on 10% increments of VAS adherence, adjusted for age, sex, and study indicator, stratified by self-reported general health status in STTR and CNICS study samples

^b^Missing *n* = 218 because some studies didn’t use the self-reported general health status item

^c^Missing *n* = 11,407 because some studies didn’t use the self-reported general health status item

### Association of ART regimens with viral suppression

Although there were no significant differences by regimen in the association of adherence with viral suppression every other regimen compared with NNRTI showed a significantly stronger association with viral suppression (Table 5). Moreover, the interaction analysis shows that both PIs (*P* < .004) and combination/other ART regimens showed significantly stronger associations than with NNRTI regimens.

**Table 5:** Associations of Adherence^d^ with Log- viral load, Stratified by ART Type among Criminal Justice-Involved (STTR) and Routine Clinical Care (CNICS) Study Samples^a^

	Mean VAS^c^	Coeff	95% CI	*P*-value	Interaction *P*-value^1^
STTR *N* = 234^b^					
STTR Overall	87	−0.17	− 0.37,0.03	0.09	NA
NNRTI (*n* = 72)	86	−0.16	−0.47, 0.14	0.30	Ref
PI (*n* = 125)	89	−0.21	−0.52, 0.10	0.18	0.82
INSTI (*n* = 12)	87	0.55	−0.43, 1.53	0.27	0.17
Other/Combo (*n* = 25)	78	−0.31	−0.77, 0.14	0.18	0.60
CNICS *N* = 11,698					
CNICS Overall	92	−0.42	−0.45, − 0.38	< 0.001	NA
NNRTI (*n* = 3347)	94	− 0.28	− 0.35, − 0.21	< 0.001	Ref
PI (*n* = 2979)	90	− 0.42	−0.49, − 0.35	< 0.001	0.004
INSTI (*n* = 2626)	93	− 0.35	− 0.42, − 0.27	< 0.001	0.20
Combination/Other (*n* = 2746)	91	− 0.51	−0.58, − 0.43	< 0.001	< 0.001

^1^Interaction *P*-value for the test of whether the regression coefficient for the given ART type is different than that for NNRTI

^a^Linear regressions of Log VL on adherence, adjusted for age, sex, and study indicator, stratified by ART type in the criminal justice and routine care samples

^b^*n* = 180 had missing ART type

^c^Mean VAS was not significantly different by regimen in STTR (*p* = 0.08), and was significantly different by regimen in CNICS (*p* < 0.001)

^d^Adherence measured in 10% increments of the VAS

## Discussion

Among criminal justice-involved persons living with HIV from seven criminal justice-focused studies in STTR, we found consistent associations between higher self-reported ART adherence, using a variety of approaches, with lower VL levels. We compared these findings to people living with HIV in routine clinical care from seven CNICS sites across the US and found similar patterns of association. In addition, the coefficients reflecting the strength of the association were generally in the same direction and of similar magnitude to those among people living with HIV in the routine clinical care sample. These findings have important implications for the care of people living with HIV who cycle through criminal justice settings, and the study of ART adherence in the continuum of HIV care among criminal justice-involved populations. Because criminal justice-involved populations, particularly those recently released from incarceration, are highly transient and hard-to-reach because of frequent unstable housing, substance use, and mental health problems they face greater challenges to ART adherence as well as to accessing HIV care [35–37]. Suitably, the findings support the use of a simple, VAS measure to assess self-reported ART adherence in criminal justice-involved populations. Its brevity, ease of administration, and interpretation make it attractive for use with low literacy populations such as the criminal justice-involved people living with HIV [13].

Of particular note, in the criminal justice sample we found that the associations of self-reported adherence with VL were robust in that there was a significant association of high levels of adherence with lower levels of VL, measured in a variety of ways – continuous (log-VL) and dichotomous. It is clinically useful to know that every 10-point increment on the 0–100% adherence scale is associated with approximately a 25–30% decrement in log-VL. The GAM analysis suggests that this association was not significantly different in magnitude at the low or high end of the VAS adherence scale. Together these findings mean that assessing self-reported ART adherence could be useful in detecting patients who are most likely to have uncontrolled viremia at the low end, as well as in detecting those who are likely well controlled at the high end of adherence reports. Comparisons of these associations with the routine care sample provided strong confirmation of the findings in the smaller criminal justice sample because in almost every analysis, the coefficients were of very similar direction and magnitude as those in routine care.

The examination of potential effect modification by CD4 cell count and general health status also enhanced the clinical relevance of our findings. In the criminal justice sample, the regression coefficients relating high levels of ART adherence with lower levels of VL were significant in both strata of CD4 count, and the interaction testing difference in the association by CD4 level was not significant, suggesting that the relationship held regardless of stage of illness. However, the point-estimate of this regression coefficient among those with CD4 level < 500 was twice as large as that among those with CD4 level ≥ 500. Furthermore in the routine care sample, the point-estimate of this regression coefficient among those with CD4 level < 500 was more than three times as large as that among those with CD4 level ≥ 500. Thus, with the large sample size the interaction testing differences in the association by CD4 level was highly significant. It is important that the association was strongest among those with the most advanced disease, and for whom clinicians would be most concerned. Nonetheless, the regression coefficients relating high levels of ART adherence with lower levels of VL were significant in both strata of CD4 count in the routine clinical care sample, supporting the robustness of the association.

Examination of the associations by self-reported general health status, however, did reveal more variation. Among the criminal justice sample, we found that the regression coefficients relating high levels of ART adherence with lower levels of VL was strong and highly significant among those with low self-reported general health status levels. However, the corresponding coefficient was quite weak (nearly zero) and non-significant among those with high self-reported health status levels. Thus, the interaction testing of the difference in the association by self-reported general health status level was highly significant. This finding supports the association among the most symptomatic, but not among those in best self-perceived health. Interestingly, the same pattern was not observed in the routine care sample. There we found the association of VAS adherence with VL was significant and of similar magnitude regardless of self-reported general health status levels, so the interaction was not significant. Moreover, we found that adherence was associated with viral suppression, especially for combination ART medications.

As with all studies, these analyses had several limitations. First, this study was cross-sectional and hence we can only report associations and cannot demonstrate causality. We restricted the criminal justice and routine clinical care samples to only those whose 30-day adherence report window covered the VL test date. While this restriction reduced the sample size substantially, whether the VL overlapped with the adherence timeframe would most likely impart random error, rather than systematic bias in one consistent direction. Inherent to smaller sample size is the reduced power of some analyses, especially the interaction or moderation analyses, such as those we conducted by CD4 and self-reported general health status. As often occurs with secondary data analyses, the data were collected for other purposes and hence did not always align with the design needs of this analysis. The smaller sample size resulting from our attempts to eliminate error reduced our power to detect associations and interactions, widening confidence intervals around the coefficients of associations. The sample size was also smaller for analysis by CD4 count and general health status than that for other variables, so we had limited power for these moderation analyses. We lacked data on barriers to adherence, or objective measures such as MEMs, pill count or number of pills in each ART regimen to make comparisons with the VAS. Despite these limitations in the data we do have viral load data that a strong anchor for adherence for this analysis. Last, although this study includes several major HIV epicenters around the US, and both jail and prison settings, the findings may not generalize to all criminal justice settings, nor to all major metropolitan or smaller areas. It also may not generalize to cases where the time frames of the VAS adherence and VL measures do not match.

## Conclusions

Overall, we found that relationships between adherence and VL, using a variety of approaches among criminal justice-involved PLWH, were robust and similar to those in routine clinical care. Consequently, the VAS adherence measure is a convenient, valid and useful adherence measure to support treatment for criminal justice-involved people living with HIV who are vulnerable to falling out of care. While we recognize that obtaining VL is essential to assessing the outcomes of care, the VAS adherence measure is useful to clinicians in this situation, where it is often difficult to measure VL regularly. It can provide results more quickly and efficiently for clinical decision-making than more complex adherence measures, or VL tests which often take days to produce results. Findings from this assessment when adherence is suboptimal (<=95%) can direct clinical care in two important ways: 1) providers can quickly intervene to optimize adherence and possibly avoid virologic failure sooner than they could if they waited for VL test results and 2) proactively order HIV-1 genotyping to assess for virologic resistance to current medications. The latter is indicated because the risk of resistance is greater with poor or intermittent adherence that often attends criminal justice-involved people living with HIV who have prevalent substance use disorders and cycles of incarceration and release. This study also provides further evidence of the validity of the VAS adherence measure for use in other survey research. This is, particularly the case in post-release criminal justice-involved populations or other situations where more objective forms of adherence measurement and more frequent VL testing are not feasible. While some research has supported the notion that criminal justice-involved populations can achieve HIV continuum of care milestones as well as those of non-criminal justice-involved populations [38], it is generally recognized that criminal justice-involved populations are at particular risk of lacking HIV care and adherence to ART [7, 39–41]. Gaps in care and loss of viral control often occur after release, when it would be impractical and cost-prohibitive to measure VL frequently or to monitor adherence with resource-intensive approaches, such as MEMS-caps [42, 43]. Thus, our finding that the magnitude of association between adherence to ART and VL was quite comparable to that in a sample of people living with HIV in routine clinical care was reassuring of the usefulness and robustness of the VAS adherence measure for use in other low resource settings. Future research should further examine the performance of adherence measures in additional hard-to-reach, disadvantaged populations.

## Data Availability

The data are available through the data-coordinating center for the STTR project (https://www.uwchscc.org/ and https://sttr-hiv.org/cms). All data requests must be approved by the STTR publications and presentations committee due to the sensitive nature of the project involving participants with substance use, HIV infection, and/or criminal justice involvement.

## Abbreviations

ART: Anti-retroviral treatment
CD4: Cluster of differentiation 4
CI: confidence interval
CNICS: Centers for AIDS Research Network of Integrated Clinical Systems
DHHS: US Department of Health and Human Services
GAMs: generalized additive models
HIV: human Immunodeficiency virus
IRB: Institutional review board
MEMS: medication event monitoring system
MSM: Men who have sex with men
NNRTIs: non-nucleoside reverse transcriptase inhibitors
OR: odds ratio
RCT: Randomized controlled trial
SD: Standard deviation
STTR: Seek, test, treat and retain
UCLA: University of California, Los Angeles
UPC: unannounced pill counts
VAS: visual analogue scale
VL: Viral load
